# Oral Health and Lifestyle Factors in Rural Adults of Jharkhand, India

**DOI:** 10.1155/2024/9100665

**Published:** 2024-02-07

**Authors:** Sandeep Kumar, Anubhuti, Abhishek Gautam, Ambar Khan, Anita B, Punit Karmacharya

**Affiliations:** ^1^Department of Public Health Dentistry, Dental Institute, RIMS, Ranchi-09, India; ^2^Conservative Dentistry and Endodontics, Ranchi, Jharkhand, India; ^3^Department of Periodontics, Government Dental College, Nalanda, Bihar, India; ^4^Department of Public Health Dentistry, College of Dental Science and Hospital, Indore, India; ^5^Department of Public Health Dentistry, Subbaiah Institute of Dental Science, Shimoga, Karnataka, India; ^6^Public Health Dentist, Department of Dentistry, B.P. Eye Foundation, Lokhathali-1, Bhaktapur, Nepal

## Abstract

**Background:**

There is a lack of health care facilities and poor oral health awareness among the rural adult population of Jharkhand which may significantly influence oral health status and lifestyle scores.

**Aim:**

To assess the oral hygiene status, lifestyle factors, and various risk factors associated with poor lifestyle scores in the rural adult population of Jharkhand.

**Materials and Methods:**

This cross-sectional study included 400 rural adults (35–44 years) populations. Face-to-face interviews were used to collect sociodemographic data and data on oral hygiene practices. Lifestyle factors were assessed using Health Practice Index (HPI). Oral health status was assessed using the oral health assessment proforma provided by the World Health Organization (WHO).

**Results:**

A significantly higher (*p* value < 0.0001) prevalence of tobacco consumption was reported by males (94.0%) compared to females (4.0%). The males (54.0%) reported significantly higher (*p* value < 0.0001) poor lifestyle scores compared to females (38.0%). A significantly higher (*p* value < 0.0001) number of oromucosal lesions (13.0%) was found in males compared to females (1.0%). There was a significant difference (*p* value < 0.0001) in the oral hygiene status between males and females with majority of males (60.0%) having poor oral hygiene. A bivariate analysis was performed, and unadjusted odds ratio was computed. The factors that became significant were then entered into logistic regression model (enter method). The results of logistic regression analysis showed that education (OR = 0.3, *p* value = 0.003), systemic diseases/long-term medication (OR = 2.9, *p* value = 0.004), tobacco consumption (OR = 2.9, *p* value = 0.006), oral hygiene status (OR = 2.4, *p* value = 0.007), and dental caries (OR = 2.9, *p* value = 0.004) were significant predictors of poor lifestyle scores.

**Conclusion:**

The rural adult population in Jharkhand has poor oral hygiene status and poor lifestyle scores. It is important to raise awareness regarding good oral hygiene and the negative effects of tobacco consumption. The dental visit should be encouraged, and the concept of preventive care needs to be instilled.

## 1. Introduction

An individual's “oral health is a significant indicator of their overall health.” A clinical examination of “dental caries, periodontal disease, tooth loss, and unmet dental treatment needs is the most common method for measuring oral health in epidemiological research” [1]. Among the many problems associated with poor oral hygiene are periodontitis, tooth decay, pain and discomfort, infection, and tooth loss [2].

Several factors influence oral health. Lifestyle factors are one of them. Living style is a general pattern of behavior determined by social, cultural, and individual characteristics as well as living conditions [3]. There are disparities between rural and urban populations in the status of their oral health [4].

The people residing in rural areas of Jharkhand may have different lifestyle factors and oral hygiene status. The rural populations in Jharkhand have been deprived of oral health care services. They still adhere to traditional oral practices [5]. There are a lack of access to oral health care centers, financial issues, and lack of awareness due to which there is a heavy burden of oral diseases in rural areas. It is unclear how the changes in lifestyle factors have affected the oral health status of the rural population. Although previous studies assessing oral health status in rural population or lifestyle factors in rural population have been done, their association has never been explored, which prompted the authors to undertake this study. The assessment of lifestyle factors is done using the Health Practice Index (HPI) which is a valid instrument for assessing lifestyle factors of an individual and used worldwide [6, 7]. This Index includes eight health practices recommended by Morimoto, namely, exercise, alcohol consumption, smoking, sleeping hours, nutritional balance, eating breakfast, working hours, and subjective stress [6]. For evaluating the oral hygiene, the oral hygiene index simplified was used, which has been used worldwide in a number of studies [8, 9]. The lifestyle factors and oral hygiene status and their association have never been studied in the rural adult population of Jharkhand. Hence, this study aimed to assess the oral health status, which includes evaluation of dental caries, periodontal diseases, malocclusion and oromucosal lesions, and lifestyle factors as assessed by HPI in the rural adult population of Jharkhand. The study also explored for various factors associated with poor lifestyle scores in the rural adult population of Jharkhand. The potential limitation of this study was that it was carried out on adults only, and hence, the results cannot be generalized. Also, it included only rural population, and therefore, a comparison with urban population could not be carried out. The strength of the study was that it included a comprehensive examination of the oral cavity, and common oral diseases like dental caries, periodontal diseases, malocclusion, and oromucosal lesions were assessed. Lifestyle factors were evaluated using validated Health Practice Index. Also, the rural population contribute to nearly 70% of the population residing in Jharkhand, and hence, assessing their lifestyle scores and oral health status provides a clear idea of residents in Jharkhand and broadly contributes to oral health data bank of India. This will also enable policy makers to draft effective policies for improvement in the lifestyle factors of rural adult population, plan health awareness campaigns, and oral health camps in these unreached locations. The dental institutions can adopt these villages and work for oral health promotion of these deprived people. Tobacco awareness campaigns can be planned, and audio–visual aids for creating oral health awareness and improving the oral hygiene practices of these people can be scheduled.

## 2. Materials and Methods

We conducted a cross-sectional study on adults (aged 35–44 years) residing in the Bero Block which is a rural area of Jharkhand. It took 4 months to complete this study (Jan 2023–April 2023). The study received ethical approval to be conducted from the institutional ethics committee of Rajendra Institute of Medical Sciences, Ranchi, with IEC clearance number 282.

In total, 400 adults participated in this study. “Based upon the findings of the pilot study and using the formula recommended by World Health Organization (WHO) for sample size calculation” [10], it was found that a minimum number of 374 would be sufficient. This was rounded off to 400. There was an equal representation of males and females.

The individuals fulfilling the required adult age criteria (35–44 years age), residing in Bero block for atleast 10 years, willing to participate, and signed the informed consent were included in this study. The study's objectives were explained to the participant, and those not willing to participate or were medically compromised were excluded. Also, those individuals who were not fulfilling the required age criteria or were not permanent residents of Bero block were excluded.

An informed consent was sought from the participants in their local language prior to the conduct of the study. The consent form was drafted as per the guidelines recommended by the institutional ethics committee of RIMS, Ranchi. The consent form clearly mentioned that the study objectives have been explained to the participants and that they are voluntarily giving their consent to participate in the study and that they can withdraw from the study at any point of time without giving any reason and without penalty or loss of routine care benefits.

We developed a questionnaire to collect information about participants' sociodemographic characteristics and oral hygiene practices. We assessed lifestyle factors using the Health Practice Index (HPI) which has been used universally and is a reliable and valid tool for assessing the lifestyle factors as reported by a number of studies [6, 7]. This Index comprises eight questions which evaluates an individual exercise patterns, alcohol habits, smoking behavior, sleeping patterns, nutritional uptake, breakfast habits, working duration, and subjective stress. The results are evaluated on a 2-point scale where “good health practices are coded as 1 and poor health practices are coded as 0.” Thus, the minimum and maximum scores belong to the range of 0–8. Based upon Morimoto's criteria, lifestyle was further divided into poor (0–3), moderate (4–5), and good (6–8) [11].

The assessment of oral health status was carried out using the WHO oral health assessment proforma [10]. This oral health assessment proforma comprises different sections to assess oral health. This proforma was recommended for use by the World Health Organization in 1997 and is a reliable and valid tool for assessing oral diseases and has been used in a number of previous researches conducted worldwide [12, 13]. The common oral diseases like dental caries, oromucosal lesions, periodontal diseases, malocclusion, and others can be evaluated using this proforma.

The assessment of oral hygiene status was done as per the guidelines of the OHI-S Index given by Greene and Vermilion [14]. A total of six surfaces of six different teeth are examined separately for debris and calculus score using recommended instruments like mouth mirror, light source, and Number 23 explorer. The debris and calculus scores obtained are then added to obtain the oral hygiene index simplified score. The oral hygiene index is used universally to assess the oral hygiene status of the population and is a reliable and valid index for evaluating oral hygiene [8, 9]. All instruments were sterilized before use, and strict infection control protocols were followed throughout the study.

The participants were selected using a stratified random sampling technique. For the selection of the study participants, the Bero block was divided into four zones. From each zone, two places were randomly selected using lottery method. From each of the identified places in every zone, the participants were randomly selected. A total of hundred participants were selected from each zones taking care to include equal number of males and females in the study. A total of 10–15 adults were examined every day by a trained investigator (dentist) along with the help of a trained assistant. Face-to-face interviews were conducted to collect sociodemographic information, oral hygiene practices, and other information. All doubts were clarified there. The incomplete forms were removed.

All statistical analysis was done using SPSS V 20. Frequency distribution analysis and *χ*^2^ test were performed. The comparison of sociodemographic data, oral hygiene practices lifestyle factors, and other oral health parameters between males and females were done using *χ*^2^ test and *p* values were computed. To identify factors associated with poor lifestyle scores, bivariate analysis was followed by logistic regression analysis (enter method) was selected. In the bivariate analysis, the poor lifestyle scores were kept as dependent variable, whereas the various sociodemographic correlates, oral hygiene practices, dental visits, and other oral health parameters were considered as independent variable. The unadjusted odds ratio and *p* value were first determined. The factors that became significant were then entered into the regression model using enter method and adjusted for confounding factors. In the regression analysis, adjusted odds ratio and *p* values were computed after adjusting for confounders. In the present study, the factors that remained significant after adjusting for confounders were considered as actual predictors for poor lifestyle scores. Statistical significance was determined by *p* value < 0.05.

## 3. Results

The sociodemographic data, oral hygiene practices, and other associated factors as evident from Table 1 were collected to have an overview of study participants taking part in the research study. This collected information was later used to predict factors associated with poor lifestyle scores, which was one of the aims of this study. There was no significant difference observed in education (*p* value = 0.920), occupation (*p* value = 0.840), use of oral hygiene aid (*p* value = 0.839), material for oral hygiene maintenance (*p* value = 0.839), frequency of toothbrushing (*p* value = 0.055), use of mouthwash (*p* value = 0.239), suffering from systemic diseases or use of long-term medication (*p* value = 0.841), and previous dental visits (*p* value = 0.635), between males and females of Bero block. However, there was a significant difference (*p* value < 0.0001) observed in the habit of tobacco consumption with males (94.0%) reporting a higher prevalence of tobacco consumption compared to females (4.0%) (Table 1).

The assessment of the lifestyle score among the rural adult population of Jharkhand was one of the main aims of the study. The Health Practice Index was used to compute the lifestyle scores. It was categorized into good, fair, and poor. It was found that 6.0% males showed good, 40.0% showed fair, and 54.0% showed poor lifestyle scores. On the contrary, 32.0% females showed good, 30.0% showed fair, and 38.0% showed poor lifestyle scores. A significant difference (*p* value < 0.0001) was found in the lifestyle scores between males and females with males (54.0%) reporting a significantly higher poor lifestyle score compared to females (38.0%) (Table 2).

The aim of the study was also to evaluate oral health status for which WHO oral health proforma was used. The common oral diseases like dental caries, periodontal diseases, malocclusion, and oromucosal lesions were assessed. The oral hygiene was assessed using OHI-S Index. The oral health findings were later entered into the regression model to identify predictors for poor lifestyle scores. There was a significant difference (*p* value < 0.0001) observed in the oral hygiene status between males and females. It was observed that 12.0% of the males reported good, 28.0% fair, and 60.0% poor oral hygiene status. On the contrary, 38.0% females reported good, 32.0% reported fair, and 30.0% reported poor oral hygiene status. Also, there was a significant difference (*p* value < 0.0001) observed in the oromucosal lesions between males and females. It was found that 13.0% of the males have developed the lesion, whereas only 1.0% of the females had developed the lesion. No significant differences were found in development of caries (*p* value = 0.417), malocclusion (*p* value = 0.813), and periodontal diseases (*p* value = 0.088) between males and females (Table 3).

Besides the evaluation of oral health status and lifestyle factors, the study also explored for various factors associated with poor lifestyle scores in the rural adult population of Jharkhand. To identify the predictors of poor lifestyle scores, a bivariate analysis and a logistic regression analysis using enter method were conducted. It was found that, out of the various variables tested, five variables, namely, education, systemic diseases/long-term medication, tobacco consumption, oral hygiene status, and dental caries became significant. The individuals who were illiterate (OR = 3.3, *p* value = 0.003), suffering from systemic diseases or under long-term medication (OR = 2.9, *p* value = 0.004), and having habit of tobacco consumption (OR = 2.9, *p* value = 0.006) were more likely to have poor lifestyle scores. Also, individuals who had poor oral hygiene status (OR = 2.4, *p* value = 0.007) and were suffering from dental caries (OR = 2.9, *p* value = 0.004) were more likely to have poor lifestyle scores (Table 4).

## 4. Discussion

The present study assessed oral health status, lifestyle scores, and factors contributing to poor lifestyle scores in the rural adult population of Jharkhand. The assessment of lifestyle scores was done as per Morimoto's criteria [11] with males having a significantly higher poor lifestyle score compared to females. Similar findings have been reported in the study done by Fiala and Brazdova [15]. The authors found that the prevalence of adverse habits, poor dietary habits, and less attention to physical activity were significant contributors to poor lifestyle scores in men. Hence, this implies that these lifestyle determinants need to be uplifted targeting the male population in rural areas.

An interesting finding in the study was that more than two-third of the study population had never visited a dentist before. Several studies conducted in different parts of India have shown that the Indian populations rarely visit a dental surgeon for preventive oral health checkups [16, 17]. Also, the study revealed that the use of mouthwashes was not very popular among the rural adult population in Jharkhand. It has been found that chemical plaque control in adjunct to mechanical aids is superior in plaque control and oral hygiene maintenance [18].

Males reported a significantly higher poor oral hygiene status compared to females in rural areas, which are similar to the findings reported by Deolia et al., [19]. This implies that males have poor oral hygiene practices and poor oral health awareness compared to females. Educational interventions are needed for oral health upliftment in males residing in rural areas. Also, more than two-third of the males reported not visiting the dentist regularly which may be a plausible reason for poor oral hygiene found in males. The visit to the dentist needs to be encouraged not only for curative reasons but also for preventive purposes. In a study done by Karuveettil et al., [20] gender proved to be a risk factor for oral diseases in India, with comparable metadata of other countries. By identifying gender-based risk groups for oral diseases, interventions or effective strategies can be developed to control and prevent these diseases [20].

Tobacco contains a potentially harmful substance called nicotine and various other carcinogenic compounds, and many Indian studies have reported that “consumption of tobacco leads to the development of oral mucosal lesions and oral carcinoma” [21]. The males reported a prevalence of 13% oromucosal lesions compared to females in which only 1% oro mucosal lesions were found. A higher prevalence of oromucosal lesions have been reported in males in studies done by Bhatnagar et al. [22] and Mathew et al. [23]. It is believed that the tobacco-associated habits being more prevalent in males lead to higher predominance of development of oromucosal lesions in males. It thus implies that habituated patients must be motivated to quit smoking and other adverse habits. It is recommended to perform scaling as a motivation tool to quit tobacco in such patients. The tobacco intervention programs need to be started in rural areas targeting the male population. Also, awareness needs to be created related to harmful consumption of tobacco and adopt better oral hygiene practices.

Logistic regression analysis revealed that illiterate individuals were more likely to have poor lifestyle scores than literate individuals. Similar findings have been reported in the study done by Foster et al. [24] and Zhang et al. [25]. Oral diseases are caused by a range of modifiable risk factors common to many noncommunicable diseases (NCDs), including sugar consumption, tobacco use, alcohol use and poor hygiene, and their underlying social and commercial determinants. Various studies have found that there is an association of different components of Health Practice Index individually with the oral health outcomes [21, 26]. However, this study is different from other studies because this study generated a composite score of various factors that are known to affect the oral health outcomes, thereby providing a comprehensive picture by taking into consideration every determinant of lifestyle factors which have been reported in literature to affect oral health outcomes. The authors believe that, if an individual is educated, then he has more awareness toward practicing a healthy lifestyle and adopting healthier lifestyle practices has a positive impact on oral health status. An improvement in the individual determinants of lifestyle factors will significantly have a positive influence in the oral health outcomes.

As compared to healthy individuals, people with systemic diseases had a poorer lifestyle score. Several studies have found a direct association between various systemic illnesses with poor lifestyle scores [27, 28]. The authors think that individuals suffering from systemic diseases are more likely to have an altered dietary pattern and less physical activity which is likely to reduce their lifestyle scores.

The individuals who were having poor oral hygiene status were having poor lifestyle scores. It is believed by the authors that poor oral hygiene leads to a “poor quality of life-related to oral health” [29]. In addition to this, the authors believe that poor oral hygiene status as reported in this study may be a sequel to adverse oral habits like tobacco consumption and poor oral hygiene practices.

The individuals who were having dental caries were having poor lifestyle scores. Dental caries is often associated with “foul smell, pain and impairs oral health-related quality of life” [30]. This directly affects sleep, exercise, and other day-to-day activities which lead to lower lifestyle scores.

Tobacco consumption was found to be more prevalent among males compared to females. The individuals who were having the habit of tobacco consumption were more likely to have a poor lifestyle score. Tobacco consumption significantly impairs sleep, eating habits, and quality of life and has numerous systemic adverse effects which impair lifestyle scores [31].

The study findings suggest that oral health status and lifestyle factors of the rural population need to be improved. A number of intervention programs like educational interventions, balanced diet uptake, regular exercising programs, stress minimizing programs, tobacco counseling programs, and others can be prepared by drafting a comprehensive policy by the policy planners targeting the rural residents.

The study finding warrants the research to be conducted on a larger scale to evaluate the oral health and lifestyle factors of the rural adult population in India. The study findings enforce on the need for tobacco cessation interventions and interventions to improve oral hygiene practices of rural population. There are various tobacco intervention programs like complete banning the sale of tobacco products, creation of smoke free workplace, restrictions on tobacco advertising, and development of preventive programs at school level, which need to be initiated and their effectiveness to be evaluated over a period of time. There are various oral health promotion programs which can be implemented at school levels in order to improve the awareness toward oral hygiene. The concept of prevention and preventive dentistry needs to be instilled at earlier ages. This opens up avenues for a number of research that can be conducted targeting the rural population.

However, some of the limitations of the study include its “cross-sectional study design.” Although cross sectional studies are inexpensive, easy to be carried out, and provide a preliminary data for carrying further advanced studies, they do not help to establish the temporal relationship between the exposure and outcome. They only measure data at a single point of time and are thus unsuitable for studying behavior over a period of time. We used a stratified random sampling technique for selection of participants who were adults and in the age group of 35–44 years from a single rural area of Jharkhand. Hence, the generalizability of the results can be questioned. The study should have included all age groups, and more number of rural areas before the results could be generalized. Also, it should have included urban areas also so that comparison with rural and urban areas could have been done for better assessment of lifestyle differences and oral health disparities. Lifestyle scores were assessed using a standardized Health Practice Index which has eight different components which are added together to generate the composite lifestyle score. The role of individual components of lifestyle factors on oral health has not been considered in this study. Besides these, lifestyle scores may be influenced by other factors which might not have been examined in this study. For a better understanding of how lifestyle scores are influenced by various factors, longitudinal studies need to be conducted with a larger sample size, larger geographical coverage, and inclusion of more wider age categories. This will help to study the influence of lifestyle factors on oral health over a period of time.

## 5. Conclusion

The oral health status in the rural residents was found to be poor. Males reported a significantly poor oral hygiene status and more number of oromucosal lesions compared to females. No significant differences were found in the prevalence of dental caries and malocclusion between males and females. The evaluation of the lifestyle score showed that males had significantly higher poor lifestyle scores compared to females. Females had a better oral hygiene status compared to males, whereas males had a higher number of oromucosal lesions compared to females. Tobacco consumption was more prevalent among males. More than two-thirds of the study population had never visited a dentist before. Besides the primary objectives, the study also explored for possible risk factors for poor lifestyle scores for which regression analysis was performed. Logistic regression showed that five variables, namely, education, systemic diseases/long-term medication, tobacco consumption, oral hygiene status, and dental caries were the main factors responsible for poor lifestyle scores.

The rural populations contribute nearly two-third of the local population of Jharkhand. Thus, the study findings are an indirect reflection of the findings of the majority population of Jharkhand. In order to improve the health equity and focus on oral health, there is an urgent need for assessment of community needs, to bring oral care to schools, and to use technology to address the gaps in oral health between rural and urban population so that health care disparities are addressed. This will improve the overall health and overall well-being of the rural residents. The strength of the study was that it focused on the rural population only with equal representation of both the genders. Some of the bias that might affect the results of the study are information bias and memory bias. These biases were eliminated by not including such individuals who had memory disorders and double checking the responses filled by the participants. Thus, the results obtained in the present study are not affected by these biases. Thus, it is concluded that the rural population of Jharkhand has poor oral hygiene status and poor lifestyle scores. These findings indirectly demonstrate that the oral health of residents in rural areas is poor and needs urgent interventions. The policymakers should focus on oral health promotion of the rural residents. There are a number of barriers in access to oral health care, which needs to be identified and eliminated. This study has identified some of the oral hygiene practices which need to be improved by creating oral health awareness. Also, more of oral health interventions like mobile dental clinics, teledentistry, dental camps, and educational initiatives need to be planned for development of healthy oral health behaviors.

## Figures and Tables

**Table 1:** Comparison of sociodemographic characteristics, oral hygiene practices, and other associated factors between males and females.

Factor	Categories	Males number (%)	Females number (%)	*p* value
Education	Literate	102 (51.0%)	103 (51.5%)	0.920
	Illiterate	98 (49.0%)	97 (48.5%)	

Occupation	Working	111 (55.5%)	113 (56.5%)	0.840
	Not working	89 (44.5%)	87 (43.5%)	

Oral hygiene aid used	Toothbrush	120 (60.0%)	123 (61.5%)	0.839
	Finger	25 (12.5%)	27 (13.5%)	
	Other	55 (27.5%)	50 (25.0%)	

Material used	Toothpaste	120 (60.0%)	123 (61.5%)	0.839
	Toothpowder	25 (12.5%)	27 (13.5%)	
	Others	55 (27.5%)	50 (25.0%)	

Frequency of toothbrushing	≤Once daily	170 (85.0%)	155 (77.5%)	0.055
	Twice or more daily	30 (15.0%)	45 (22.5%)	

Mouthwash	Yes	12 (6.0%)	7 (3.5%)	0.239
	No	188 (94.0%)	193 (96.5%)	

Systemic diseases/long-term medication	Yes	96 (48.0%)	94 (47.0%)	0.841
	No	104 (52.0%)	106 (53.0%)	

Previous dental visit	Yes	44 (22.0%)	48 (24.0%)	0.635
	No	156 (78.0%)	152 (76.0%)	

Tobacco consumption	Yes	188 (94.0%)	8(4.0%)	0.0001^*∗*^
	No	12 (6.0%)	192 (96.0%)	

^*∗*^*p* value < 0.05: statistically significant.

**Table 2:** Comparison of lifestyle scores based upon Morimoto's criteria between males and females.

Factor	Categories	Male number (%)	Female number (%)	*p* value
Lifestyle scores	Good	12 (6.0%)	64 (32.0%)	<0.0001^*∗*^
	Fair	80 (40.0%)	60 (30.0%)	
	Poor	108 (54.0%)	76 (38.0%)	

^*∗*^*p* value < 0.05: statistically significant.

**Table 3:** Comparison of oral health parameters between males and females.

Factors	Categories	Male number (%)	Female number (%)	*p* value
Oral hygiene status (OHI-S)	Good	24 (12.0%)	76 (38.0%)	<0.0001^*∗*^
	Fair	56 (28.0%)	64 (32.0%)	
	Poor	120 (60.0%)	60 (30.0%)	

Dental caries	Present	120 (60.0%)	112 (56.0%)	0.417
	Absent	80 (40.0%)	88 (44.0%)	

Malocclusion	Present	46 (23.0%)	48 (24.0%)	0.813
	Absent	154 (77.0%)	152 (76.0%)	

Oromucosal lesions	Present	26 (13.0%)	2 (1.0%)	<0.0001^*∗*^
	Absent	174 (87.0%)	198 (99.0%)	

Periodontal disease	Present	186 (93.0%)	176 (88.0%)	0.088
	Absent	14 (7.0%)	24 (12.0%)	

^*∗*^*p* value < 0.05: statistically significant.

**Table 4:** Logistic regression analysis (enter method) to identify factors associated with poor lifestyle scores.

Factor	Categories	Unadjusted odds ratio	*p* value	Adjusted odds ratio	*p* value
Education	Illiterate	3.3	0.003^*∗*^	3.3	0.003^*∗*^
	Literate	1.0		1.0	

Occupation	Working	0.8	0.468	0.7	0.422
	Not working	1.0		1.0	

Oral hygiene aid used	Toothbrush	0.8	0.384	0.8	0.382
	Finger	0.9		0.9	
	Other	1.0		1.0	

Material used	Toothpaste	0.8	0.365	0.8	0.372
	Toothpowder	0.9		0.9	
	Others	1.0		1.0	

Frequency of toothbrushing	≤Once daily	0.7	0.333	0.8	0.423
	Twice or more daily	1.0		1.0	

Mouthwash	Yes	0.8	0.356	0.8	0.366
	No	1.0		1.0	

Systemic diseases/long-term medication	Yes	2.8	0.005^*∗*^	2.9	0.004^*∗*^
	No	1.0		1.0	

Previous dental visit	Yes	0.8	0.322	0.6	0.377
	No	1.0		1.0	

Tobacco consumption	Yes	2.9	0.007^*∗*^	2.9	0.006^*∗*^
	No	1.0		1.0	

Oral hygiene status (OHI-S)	Poor	2.3	0.008^*∗*^	2.4	0.007^*∗*^
	Fair	2.1		2.1	
	Good	1.0		1	

Dental caries	Present	2.8	0.005^*∗*^	2.9	0.004^*∗*^
	Absent	1.0		1.0	

Malocclusion	Present	1.5	0.234	1.4	0.254
	Absent	1.0		1.0	

Oromucosal lesions	Present	1.4	0.289	1.3	0.292
	Absent	1.0		1.0	

Periodontal disease	Present	1.6	0.111	1.8	0.344
	Absent	1.0		1.0	

^*∗*^*p* value < 0.05: statistical significant difference.

## Data Availability

Raw data will be made available to the concerned person upon request to the authors.

## References

[1] Bhat M., Bhat S., Roberts-Thomson K. F., Do L. G. (2021). Self-rated oral health and associated factors among an adult population in rural India—an epidemiological study. *International Journal of Environmental Research and Public Health*.

[2] Jahangiry L., Bagheri R., Darabi F., Sarbakhsh P., Sistani M. M. N., Ponnet K. (2020). Oral health status and associated lifestyle behaviors in a sample of Iranian adults: an exploratory household survey. *BMC Oral Health*.

[3] Baskaradoss J. K., Geevarghese A., Al-Mthen A. (2019). Influence of lifestyle on dental health behavior. *Journal of Lifestyle Medicine*.

[4] Handa S., Prasad S., Rajashekharappa C. B., Garg A., Ryana H. K., Khurana C. (2016). Oral health status of rural and urban population of Gurgaon block, Gurgaon district using WHO assessment form through multistage sampling technique. *Journal of Clinical and Diagnostic Research*.

[5] Kumar G., Dileep C. L., Sethi A. K., Gupta B. (2019). The Birhor tribes of Ramgarh district, Jharkhand—a ferret into their oral health status and treatment needs. *Medicine and Pharmacy Report*.

[6] Nakayama K., Yamaguchi K., Maruyama S., Morimoto K. (2001). The relationship of lifestyle factors, personal character, and mental health status of employees of a major Japanese electrical manufacturer. *Environmental Health and Preventive Medicine*.

[7] Kawada T., Otsuka T., Inagaki H. (2011). Relationship among lifestyles, aging and psychological wellbeing using the General Health Questionnaire 12-items in Japanese working men. *The Aging Male*.

[8] Baishya B., Satpathy A., Nayak R., Mohanty R. (2019). Oral hygiene status, oral hygiene practices and periodontal health of brick kiln workers of Odisha. *Journal of Indian Society of Periodontology*.

[9] Pizzi S., Sola M. C., Artoni L. (1988). Indagine sullo stato di salute e di igiene orale dei bambini di una scuola elementare. Stato di salute e di igiene orale in età scolare [Study on health status and oral hygiene of children at a primary school. Health status and oral hygiene in schoolchildren]. *Acta Biomed Ateneo Parmense*.

[10] World Health Organization (1997). *Oral Health Surveys: Basic Methods*.

[11] Morimoto K., Obe G., Natarajan A. T. (1990). Life-style and genetic factors that determine the susceptibility to the production of chromosome damage. *Chromosomal Aberrations: Basic and Applied Aspects*.

[12] Mohan R., Venkatanarasu B., Rao B. V., Eswara K., Martha S., Hemasundar H. (2019). Assessment of oral health status and dental treatment needs among 12- and 15-year-old school-going children of fisherman community residing at east coast road, Chennai: a cross-sectional study. *Journal of Pharmacy And Bioallied Sciences*.

[13] Al-Otaibi M. F., Al-Mamari F., Baskaradoss J. K. (2012). Oral health status of 12-year-old school children in Yemen. A cross- sectional survey. *European Journal of Paediatric Dentistry*.

[14] Greene J. G., Vermillion J. R. (1964). The simplified oral hygiene index. *The Journal of the American Dental Association*.

[15] Fiala J., Brázdová Z. (2000). A comparison between the lifestyles of men and women—parents of school age children. *Central European Journal of Public Health*.

[16] Kumar S., Debnath N., Ismail M. B. (2015). Prevalence and risk factors for oral potentially malignant disorders in Indian population. *Advances in Preventive Medicine*.

[17] Kumar S., Kumar A., Gupta A., Singh S. K., Gupta A., Mehta P. (2021). Assessment of the relationship between oral health behavior, oral hygiene, and gingival status of adolescent tobacco consumers in Ranchi, Jharkhand: a comparative study. *Advances in Preventive Medicine*.

[18] Vyas T., Bhatt G., Gaur A., Sharma C., Sharma A., Nagi R. (2021). Chemical plaque control—a brief review. *Journal of Family Medicine and Primary Care*.

[19] Deolia S. G., Kela K. S., Sawhney I. M., Sonavane P. A., Nimbulkar G., Reche A. (2020). Evaluation of oral health care seeking behavior in rural population of central India. *Journal of Family Medicine and Primary Care*.

[20] Karuveettil V., Krishna K., Ramanarayanan V. (2022). Is gender a risk factor for oral diseases in India? A metadata exploration. *Public Health and Toxicology*.

[21] Jiang X., Wu J., Wang J., Huang R. (2019). Tobacco and oral squamous cell carcinoma: a review of carcinogenic pathways. *Tobacco Induced Diseases*.

[22] Bhatnagar P., Rai S., Bhatnagar G., Kaur M., Goel S., Prabhat M. (2013). Prevalence study of oral mucosal lesions, mucosal variants, and treatment required for patients reporting to a dental school in North India: in accordance with WHO guidelines. *Journal of Family and Community Medicine*.

[23] Mathew A. L., Pai K. M., Sholapurkar A. A., Vengal M. (2008). The prevalence of oral mucosal lesions in patients visiting a dental school in southern India. *Indian Journal of Dental Research*.

[24] Foster H. M. E., Ho F. K., Mair F. S. (2022). The association between a lifestyle score, socioeconomic status, and COVID-19 outcomes within the UK Biobank cohort. *BMC Infectious Diseases*.

[25] Zhang Y.-B., Chen C., Pan X.-F. (2021). Associations of a healthy lifestyle and socioeconomic status with mortality and incident cardiovascular disease: two prospective cohort studies. *BMJ*.

[26] Guasch-Ferré M., Li Y., Bhupathiraju S. N. (2022). Healthy lifestyle score including sleep duration and cardiovascular disease risk. *American Journal of Preventive Medicine*.

[27] Sayadi L., Faezi S. T., Hasanpour M., Alahmadi S. J. (2021). The relationship of lifestyle with disease activity among patients with systemic lupus erythematosus: a descriptive-correlational study. *Mediterranean Journal of Rheumatology*.

[28] Norde M. M., Fisberg R. M., Marchioni D. M. L., Rogero M. M. (2020). Systemic low-grade inflammation-associated lifestyle, diet, and genetic factors: a population-based cross-sectional study. *Nutrition*.

[29] Tefera A. T., Girma B., Adane A. (2023). Oral health-related quality of life and oral hygiene status among special need school students in Amhara region, Ethiopia. *Health and Quality of Life Outcomes*.

[30] Bukhari O. M. (2020). Dental caries experience and oral health-related quality of life in working adults. *The Saudi Dental Journal*.

[31] Vera V., Onate G., Fernández M. (2021 Jul 30). Tobacco consumption in Chilean university students and associations with anthropometry, eating habits, and sleep quality multicentric study. *Journal of Preventive Medicine and Hygiene*.

